# New insights into the role of cellular senescence and chronic wounds

**DOI:** 10.3389/fendo.2024.1400462

**Published:** 2024-11-04

**Authors:** Huiqing Yang, Xin Zhang, Bo Xue

**Affiliations:** ^1^ Institute of Evolution and Biodiversity, College of Marine Life Sciences, Ocean University of China, Qingdao, China; ^2^ College of Marine Life Sciences, Ocean University of China, Qingdao, China

**Keywords:** cellular senescence, chronic wounds, tissue repair, wound microenvironment, signal pathways

## Abstract

Chronic or non-healing wounds, such as diabetic foot ulcers (DFUs), venous leg ulcers (VLUs), pressure ulcers (PUs) and wounds in the elderly etc., impose significant biological, social, and financial burdens on patients and their families. Despite ongoing efforts, effective treatments for these wounds remain elusive, costing the United States over US$25 billion annually. The wound healing process is notably slower in the elderly, partly due to cellular senescence, which plays a complex role in wound repair. High glucose levels, reactive oxygen species, and persistent inflammation are key factors that induce cellular senescence, contributing to chronic wound failure. This suggests that cellular senescence may not only drive age-related phenotypes and pathology but also be a key mediator of the decreased capacity for trauma repair. This review analyzes four aspects: characteristics of cellular senescence; cytotoxic stressors and related signaling pathways; the relationship between cellular senescence and typical chronic non-healing wounds; and current and future treatment strategies. In theory, anti-aging therapy may influence the process of chronic wound healing. However, the underlying molecular mechanism is not well understood. This review summarizes the relationship between cellular senescence and chronic wound healing to contribute to a better understanding of the mechanisms of chronic wound healing.

## 1 Introduction

**Figure 1:**
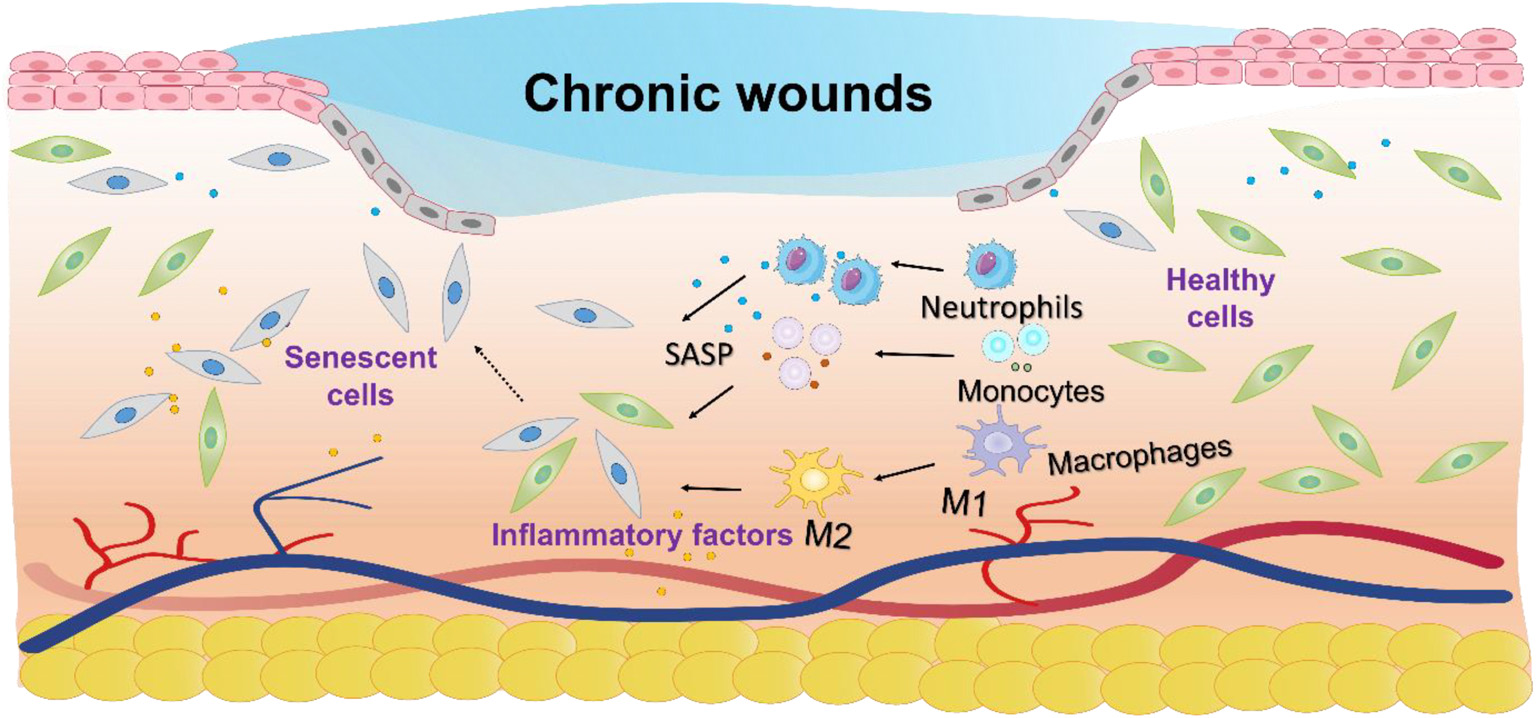
Schematic diagram of the molecular mechanisms underlying cellular senescence and chronic wounds. Cellular senescence impairs chronic wound healing by disrupting tissue repair through inflammation, altered metabolism, and apoptosis resistance.

Chronic wounds, characterized by a failure to progress beyond the inflammatory phase of normal healing, lead to prolonged low-grade inflammation, significantly reducing patient quality of life and increasing the risk of systemic complications such as infections and chronic pain (1). The incidence of chronic wounds is rising, with diabetes-related chronic wounds affecting 15 - 25% of patients (2), venous ulcers 1 - 3% (3, 4), age-related wounds 6% (5), and 16.9 - 23.8% in the hospital and intensive care population (6). There are several hypotheses regarding the difficulty in healing chronic wounds, such as the persistent state of chronic inflammation that prevents normal healing processes (7); impaired angiogenesis, which leads to insufficient blood supply to the wound (8), and the presence of biofilms that protect harmful bacteria, making infections harder to treat (9). However, no consensus has been reached among researchers to date, and the underlying mechanisms remain a subject of ongoing investigation. Increasing evidence suggests that cellular senescence plays a crucial role in the process of chronic wound healing. Senescent cells, although metabolically active, are especially harmful as they disrupt tissue repair and regeneration by altering the wound microenvironment (**Figure 1**) (10).

Cellular senescence, first described by Hayflick in 1961, is a state of stable cell cycle arrest characterized by distinct secretory properties and altered metabolism, playing a key role in aging and chronic diseases (11). Senescent cells arise in response to various stresses, such as oxidative stress and mitochondrial dysfunction, leading to cell proliferation arrest, resistance to apoptosis, and altered gene and protein expression profiles (12, 13). Proteins like BCL-2 and P53 enhance this resistance to apoptosis (14, 15). In both preclinical and clinical models, removing senescent cells through senolytic agents or therapies that inhibit the senescence-associated secretory phenotype (SASP) has shown benefits, including delaying tissue dysfunction and extending health span (16, 17). Despite these advances, chronic wound management still faces challenges like high recurrence rates and ineffective treatments (18). Recent studies suggest that cellular senescence plays a complex role in chronic wounds, though its exact mechanisms remain debated. Some evidence indicates that senescence can positively contribute to wound healing (19–21), while other studies point to its detrimental effects (22, 23). The accumulation of senescent cells, particularly in the elderly, has been linked to impaired wound healing, emphasizing the need for targeted strategies in chronic wound treatment (19, 24).

Recent advancements in the study of cellular senescence and chronic wounds underscore the necessity of a comprehensive review in this area. This article summarizes existing research, offering an overview of the relationship between cellular senescence and chronic wounds, along with the potential protective and therapeutic effects at the cellular level. By clarifying the connection between cellular senescence and wound healing, this review aims to provide valuable insights into the treatment of chronic wounds. Despite significant progress, challenges remain. Future research should prioritize uncovering the mechanisms of cellular senescence and developing innovative therapies, as targeting this process holds promise for improving chronic wound healing outcomes.

## 2 Cellular senescence

### 2.1 Characteristics of cellular senescence

**Figure 2:**
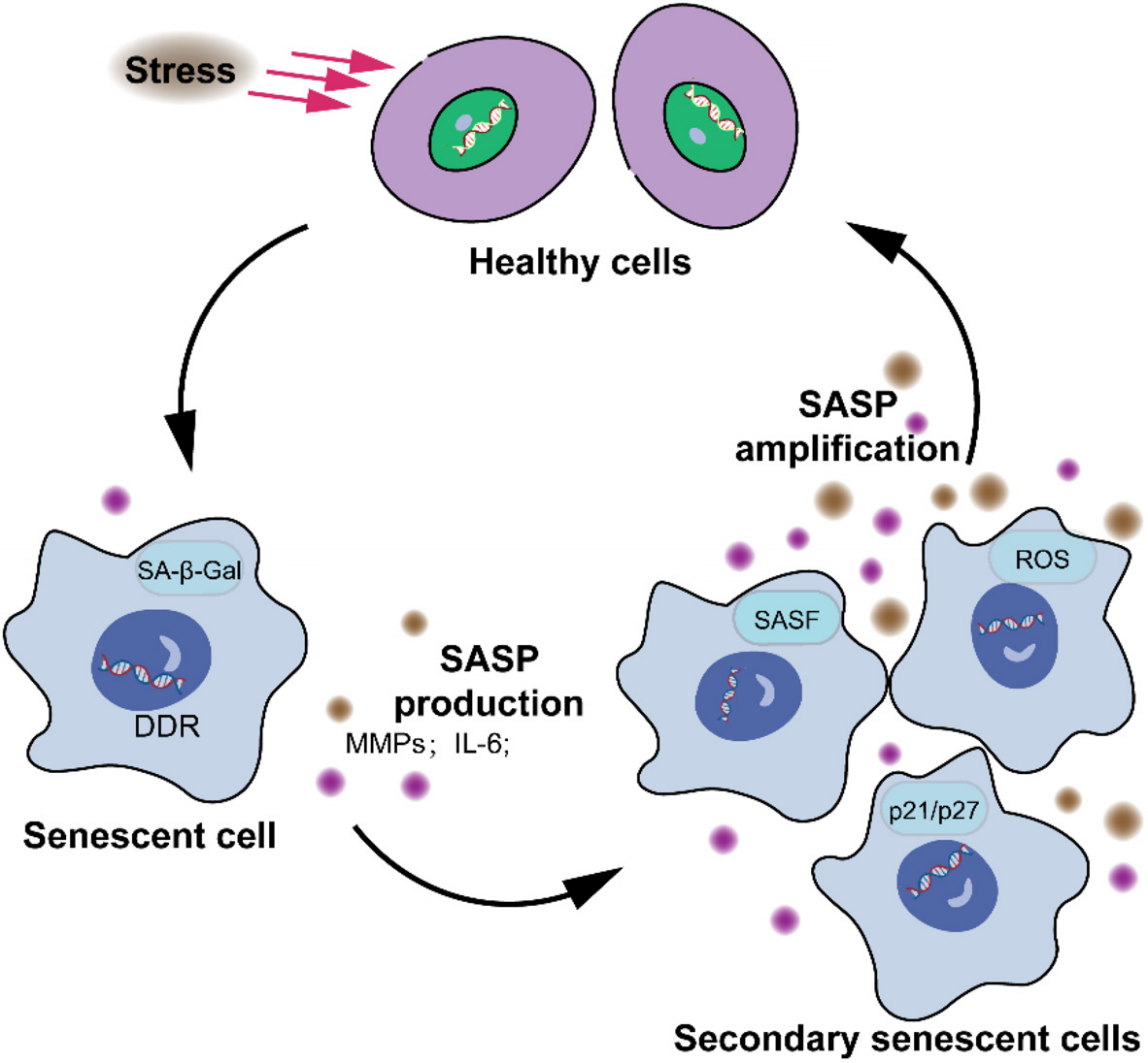
Characteristics of cellular senescence. The senescence response leads to significant alterations in cellular phenotype. These alterations encompass a largely irreversible cessation of cell proliferation, the acquisition of apoptosis resistance, and modifications in gene expression patterns. The presence or manifestation of senescence-associated markers, such as senescence-associated β-galactosidase, *p16*, senescence-associated DNA damage foci, and SAHFs, are neither consistently observed nor exclusive to the senescent state, and thus are not depicted.

Most senescent cells exhibit distinctive characteristics that diverge significantly from normal cells, encompassing morphological, biochemical, metabolic, and genetic alterations, highly stable cell cycle arrest, oxidative damage, apoptosis resistance, a SASP, and senescence-associated heterochromatin foci (SAHF), the functionality of which remains enigmatic (25–28). Studies have revealed that the retinoblastoma (Rb) family and p53 proteins play critical roles in regulating cell cycle arrest in mammals (29, 30). Moreover, genes like *p53* and *p21* are highly expressed in senescent cells. The CDK kinase inhibitors *p16* and *p21* are highly expressed in senescent cells and inhibit the activity of CDK4/6, which phosphorylates E2F to activate it as a transcription factor, thereby impeding the cell cycle process (31). The expression of *p16INK4a* increases with age and serves as a robust biomarker of senescence in both human and mouse tissues (32). The secretion of senescent cells is collectively referred to as the SASP (MMPs, inflammatory cytokines, growth modulators, angiogenic factors, etc.). SASP plays a pivotal role in the processes of cellular senescence, inflammation (33), wound healing (20), and tissue plasticity (34). However, it is unclear whether SASP links cellular senescence to organ aging (35). There are two common triggers that induce stressor-induced cellular senescence. Cellular senescence is activated by the DNA damage cascade signaling, which is defined as the DNA damage response (**Figure 2**) (DDR).

### 2.2 Cytotoxic stressors and cellular senescence

#### 2.2.1 ROS and cellular senescence

The accumulation of intracellular oxidative stress is a key feature of cellular senescence, with ROS acting as signaling molecules involved in regulating cellular metabolism (36). Excessive ROS accumulation triggers and sustains ROS-mediated senescence, also known as stress-induced premature senescence (37, 38). Studies have identified that there are various causes of chronic wounds, one of which is the excessive accumulation of ROS in the wound bed (39–41). Nuclear factor erythroid 2-related factor 2 (Nrf2) is a transcription factor that can regulate the expression of antioxidant and detoxification genes, facilitating wound healing and tissue regeneration in aging-related diseases (42). The mitochondrion, an essential organelle responsible for energy generation in eukaryotic cells, can experience dysfunction leading to cellular senescence. In senescent cells, mitochondria undergo significant changes in morphology, membrane potential, and mass (43). Mitochondrial ROS (mtROS) plays a critical role in regulating mitochondrial functions and maintaining organismal homeostasis by influencing signaling pathways, inducing mitophagy, causing mitochondrial network fragmentation, and activating antioxidant defenses (43). Interestingly, while mtROS is often associated with aging and cellular damage, it can also promote tissue repair (44–48). For instance, *Xu* et al. found that mtROS can trigger rapid and reversible mitochondrial fragmentation, facilitating actin-driven wound closure in *C. elegans* epithelial cells (49).

#### 2.2.2 High glucose-induced cellular senescence

Exogenous or endogenous HG are key mediators in the regulation of cellular senescence, serving as positive regulators of this process (50). A growing number of studies have shown that HG stimulation triggers premature cellular senescence (51–53). HG microenvironment significantly reduces the expression of miR-30a-5p in HMEC-1, increases the expression of Senescence-associated beta-galactosidase (SA-β-gal) and *p21*, promotes cellular senescence, and inhibits its proliferation, migration, and vasculogenesis (54). However, the upregulation of miR-30a-5p can effectively reverse the HG-induced senescence of HMEC-1 and improve its proliferation, migration, and vasculogenic capacity (55). Fibroblasts proliferate and migrate to the wound site and participate in the synthesis and secretion of the ECM during wound healing. Fibroblasts show a functional state during wound healing; however, the balance is disrupted in diabetic patients due to the HG microenvironment. Early studies have revealed that endothelial cell senescence takes place *in vivo* and *in vitro* (56, 57). *Hayashi* et al. found that HG exposure can induce endothelial cell senescence by decreasing the expression of eNOS protein, as indicated by the increased SA-β-gal activity (57). They observed a significant increase in the level of intracellular ROS after 3 days of HG treatment in HUVEC. L-arginine, L-citrulline, and Vitamins C and E were found to be able to mitigate this phenomenon. The potential mechanism of HG-induced endothelial cell senescence may be related to the cumulative effect of ROS.

#### 2.2.3 Inflammation induce cellular senescence

Aging-related inflammation, also known as inflammaging, shows that in the process of immune system aging, the decline of individual immune function with aging has long been regarded as the cause of inflammatory aging and body aging (58). An increasing number of studies have confirmed that immune senescence gives rise to inflammation, and older organs have higher levels of inflammatory markers (59). The inflammatory state in ulcer tissues exacerbates the delay in healing chronic wounds. This inflammation significantly affects non-specific immunity, the removal of pathogenic microbes, the elimination of dysfunctional cells, and matrix debris. Consequently, while the quantity and phenotype of leukocyte subtypes typically return to their initial levels one or two weeks after their functions are completed, the persistent inflammation continues to hinder the healing process (60). This ongoing chronic inflammation is a prominent pathological feature of chronic wounds. Due to the prolonged exposure of wounds and the inflammatory environment, the chronic wounds of DFUs are more prone to infection, causing myeloid cells, which comprise immune cells such as monocytes, macrophages, and neutrophils involved in various immune responses. Neutrophils primarily kill pathogenic microorganisms by producing reactive oxygen species (ROS), debride wounds by secreting matrix metalloproteinase (MMP)-9, phagocytose dead bacteria, and remove matrix debris through receptor-mediated endocytosis (61).

#### 2.2.4 Oncogene activation-induced cellular senescence

Another potent stressor that induces cellular senescence is oncogene activation, which was discovered by *Serrano* et al. in 1997 (62). Although the mechanisms underlying oncogene-induced senescence are complex and poorly understood, the activation of the RB and p53 pathways is necessary for the proliferative pause (63).

### 2.3 Master signaling pathways of cellular senescence

#### 2.3.1 Interleukin-6/STAT3 signaling and cellular senescence

**Figure 3:**
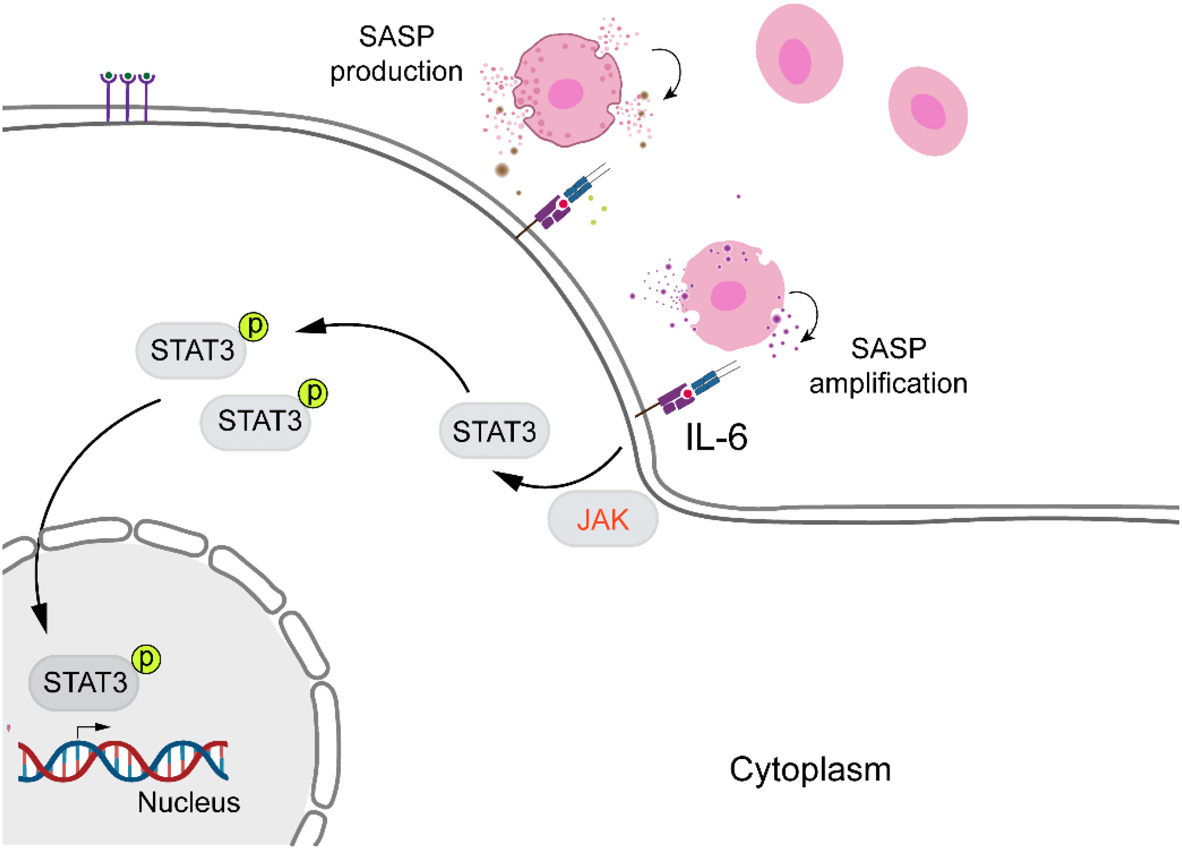
IL-6/STAT3 signaling pathway and cellular senescence. IL-6 promotes cellular senescence and age-related diseases by activating STAT3. Upon activation, STAT3 becomes phosphorylated, translocates into the nucleus, and regulates the expression of genes involved in senescence and aging.

Currently, senescence, identified as a stress response, is a key focus in wound healing research. IL-6, a cytokine with multiple physiological and pathological functions, produced by epithelial, endothelial, and fibroblast cells, is commonly found in human body fluids, which is not only closely associated with the immune system but also promotes cellular senescence and age-related diseases (33). **Figure 3** illustrates the activation of STAT family by JAKs, which is crucial for the senescence microenvironment (64). Consequently, in order to develop a valuable approach for identifying elderly individuals at risk of increased age, disease, and mortality, a recent study considered the IL-6 gene as a biomarker for frailty, regulating aging and age-related diseases (65). As mentioned earlier, *Keyes* and *colleagues* discovered that IL-6/STAT3 signaling regulates skin expression, facilitating proper wound healing in aged mice and *in vitro* (24). A similar result was obtained by *Hirotada* et al., indicating that IL-6/STAT3 can induce and maintain the senescence of human fibroblasts via ROS accumulation (66).

#### 2.3.2 The mechanistic target of rapamycin signaling and cellular senescence

**Figure 4:**
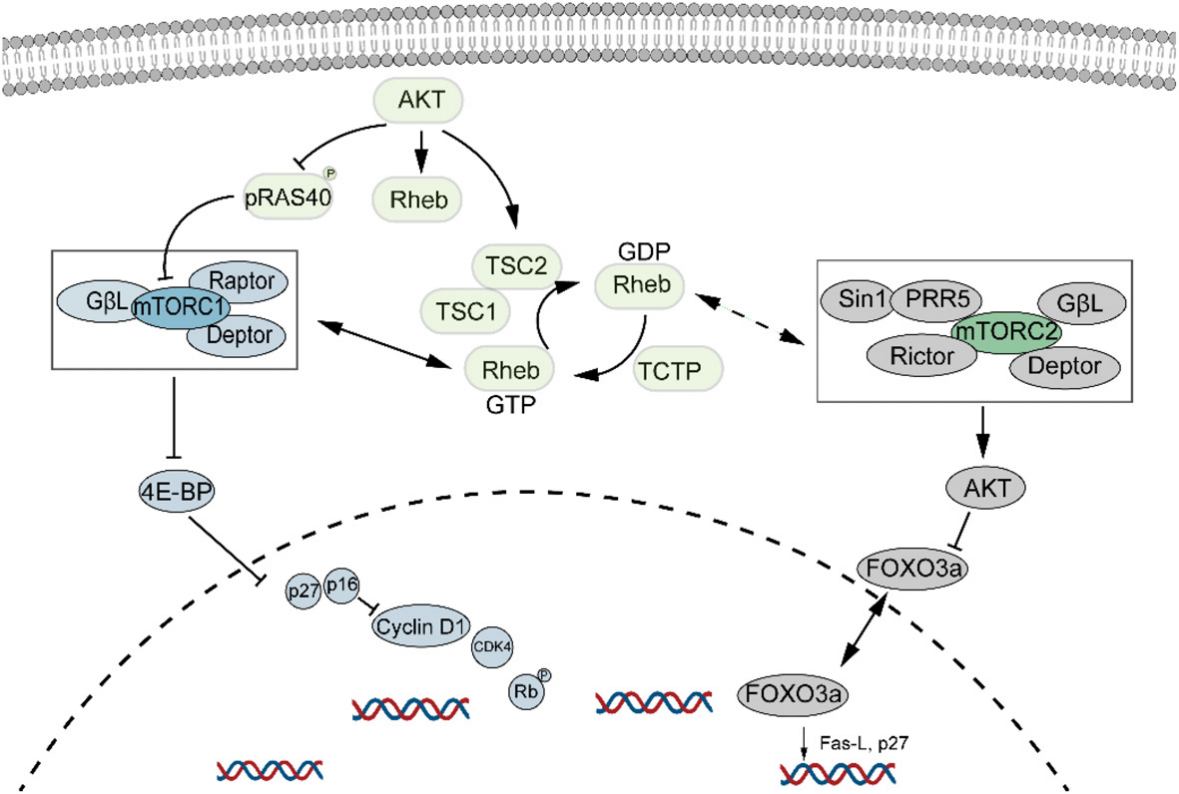
mTOR signaling pathway and cellular senescence. mTOR regulates cell growth and metabolism and is closely linked to cellular senescence. Its inhibition has been shown to extend lifespan, while promoting cellular senescence through the activation of both mTORC1 and mTORC2 pathways.

mTOR, a major regulator of cell growth and metabolism, is associated with cellular senescence (**Figure 4**). Inhibition of the mTOR pathway extends lifespan in organisms such as drosophila, yeast, and mice (67, 68). This effect may be due to dietary restriction or enhanced autophagy resulting from mTOR inhibition, which helps clear damaged proteins and organelles, thus increasing lifespan. Inhibition of mTORC1 reduces protein toxicity and oxidative stress, known stressors of cellular senescence (69). mTOR is crucial for protein synthesis, lipid, nucleotide, and glucose metabolism but is also sensitive to stressors like DNA damage and low ATP levels. Recent studies suggest that mTOR inhibitors could extend mammalian lifespan (70, 71), though potential drawbacks include glucose intolerance and immunosuppression. Nevertheless, randomized controlled trials indicate promising and safe outcomes in healthy elderly individuals (72). *Willemijn* et al. found that the mTOR pathway is relevant to both animal and human aging, with significant differential expression in seven out of forty mTOR pathway genes (73).

#### 2.3.3 Insulin-like signaling pathway and cellular senescence

**Figure 5:**
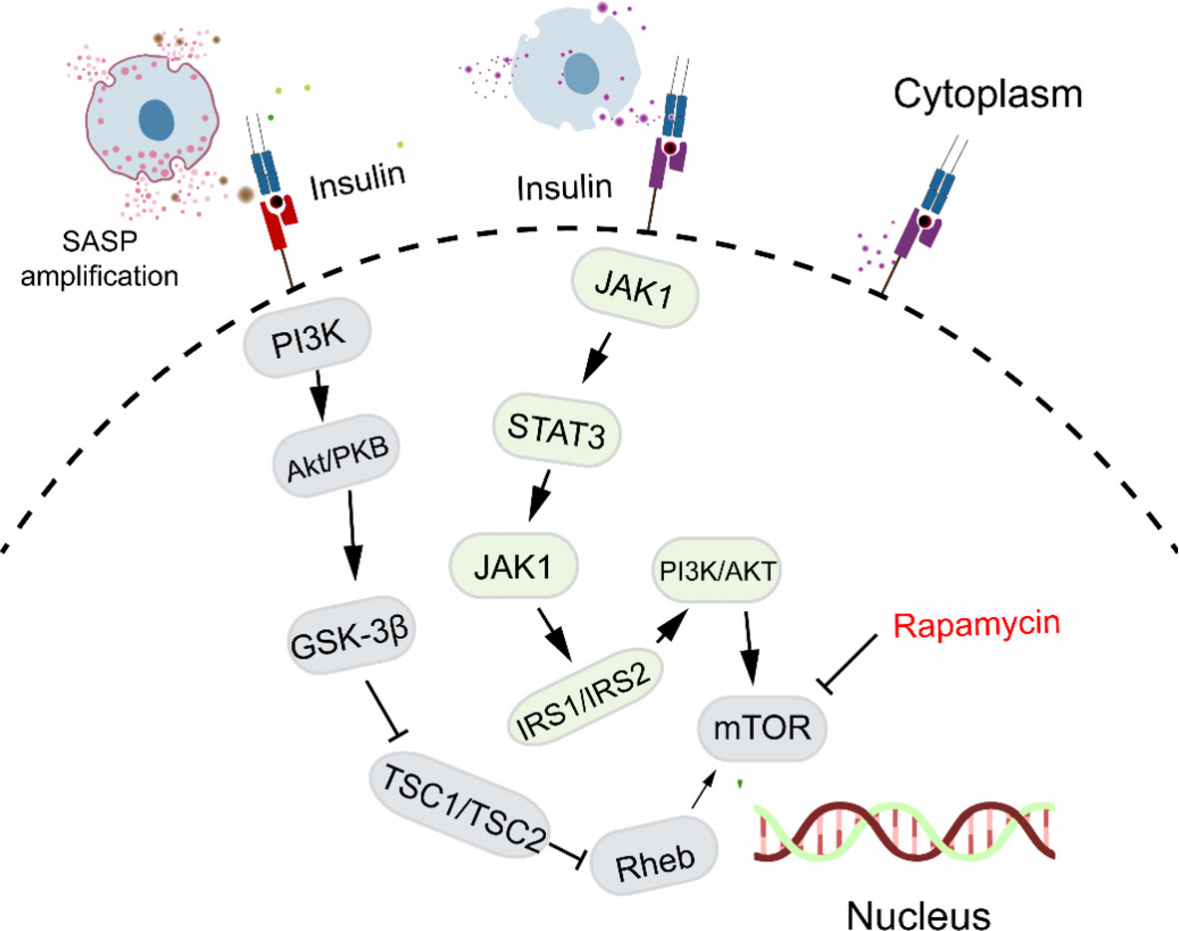
Insulin-like signaling pathway and cellular senescence. Insulin-like signaling, through Insulin-R and IGF-1R, influences aging by enhancing mTOR activity, inhibiting autophagy, and promoting low-grade inflammation linked to cellular senescence.

Insulin-like signaling is essential for development, energy balance, cell growth, and apoptosis in mammals, with Insulin-R and IGF-1R, both tyrosine kinase family members, activating pathways that include the JAK family. **Figure 5** illustrates the insulin-like signaling pathways, highlighting the roles of Insulin-R and IGF-1R in enhancing mTOR activity and inhibiting autophagy. Insulin/IGF-1 signaling also influences aging and age-related diseases through the activation of STAT3 and induction of immunosuppression (74). Currently, it is well established that insulin resistance is associated with inflammation (75, 76). Additionally, aging leads to an increase in the number of immunosuppressive M2 macrophages in various mouse tissues, including the bone marrow, spleen, lungs, and skeletal muscles (77, 78). These studies suggest that insulin signaling regulates low-grade inflammatory states that are linked to cellular senescence.

## 3 The challenges of chronic wounds

### 3.1 Pathophysiology of chronic wounds

Many chronic wounds, irrespective of their etiology, exhibit shared pathological features, notably a persistent inflammatory state that impedes progression to the proliferative phase. The accumulation of senescent cells contributes to impaired cellular proliferation and sustained inflammation, as these cells remain metabolically active but are incapable of division or tissue repair. Senescent cells secrete pro-inflammatory cytokines, exacerbating chronic inflammation. This microenvironment, characterized by elevated ROS levels, microbial burden, and unresolved inflammation, obstructs the transition from the inflammatory to the proliferative phase. In diabetic wounds, excessive infiltration of myeloid cells and a failure to transition from M1 to M2 macrophages further exacerbate the condition, leading to increased ROS production, ECM degradation, and compromised fibroblast activity (79). Understanding the evolutionary theory of aging suggests that the ability of aging tissues to maintain homeostasis and regenerate diminishes over time. Chronic wounds are marked by ECM deficiency, persistent inflammation, and dysregulated growth factor signaling, with senescent fibroblasts contributing to impaired re-epithelialization due to their resistance to apoptosis (80, 81).

### 3.2 Aging microenvironment and chronic wounds

The altered secretory phenotypes shift from an ECM synthesis phenotype to a degradation phenotype, resulting in delayed wound closure (82). The accumulation of senescent cells, driven by their resistance to apoptosis, further complicates healing, though the underlying molecular mechanisms remain poorly understood. Chronic wounds often exhibit poor responses to standard therapies, raising questions about the factors that hinder their healing. Research indicates that the presence of even 15% senescent fibroblasts in the wound microenvironment can significantly impede healing (83). Consequently, reducing the proportion of senescent cells might enhance wound regeneration (84). In one study, an anti-senescence compound was administered to diabetic chronic wounds, resulting in accelerated healing (85). Immunocytochemistry revealed that high glucose increases SA-β-gal-positive cells, highlighting the critical role of glucose metabolism dysfunction in diabetic wound healing. Conversely, other studies suggest that senescent cells may play a beneficial role in wound healing (24). For example, Chia et al. found that *p21*, *p53*, and *MMP9*-markers associated with cellular senescence—increased during the healing process in younger subjects but not in older ones (19). Velarde et al. demonstrated that epidermal SOD deficiency, linked to mitochondrial damage, enhanced epidermal differentiation and re-epithelialization, thereby accelerating wound closure in young mice. Similarly, oxidative signaling has been shown to promote epidermal wound closure (49, 86), Moreover, the SASP, particularly PDGF-AA secreted by senescent cells, has been found to aid in wound healing (20). While the exact mechanisms by which senescent cells impair tissue renewal remain unclear, it is evident that the senescence secretome may also contribute positively to wound repair. Additionally, cellular senescence has been recognized for its tumor-suppressive and anti-cancer roles.

### 3.3 The role of cellular senescence in chronic wounds

#### 3.3.1 Cellular senescence positively regulates wound healing

EMT, a process whereby epithelial cells transition to a mesenchymal state, is associated with tumor metastasis, embryo development, chronic inflammation, tissue reconstruction, and fibrosis (87–90) and plays a crucial role in wound healing, particularly in the tissue remodeling process (91). Over the past two decades, studies have identified numerous cytokines that are linked to the occurrence and progression of EMT (92–94). Some cytokines, such as the TGF-β superfamily, have increased expression that can induce the EMT process and alter the polarity of the cell. *In vitro* studies have demonstrated that TGF-β2, IL-1β, and BMP (typical senescence-related cytokines) that cause EMT can induce the upregulation of Snail expression, a master EMT transcript factor. A study found that the TGF-β–Slug signaling pathway establishes an EMT-like process that promotes the proliferation of fibroblasts and the differentiation of keratinocytes in wound healing (95, 96).

#### 3.3.2 Delayed healing through prolonged inflammation and impaired proliferation in chronic wounds

Wound healing is a complex and well-orchestrated process involving the participation and coordination of multiple cells, growth factors, and the ECM. It encompasses three overlapping phases: inflammation, proliferation, and remodeling. Dysfunctional inflammation is a hallmark of chronic wounds, which are typically characterized by a persistent low-grade inflammatory response (97). In immune surveillance, resident T cells are activated, and the infiltration of macrophages, monocytes, and neutrophils occurs during the inflammatory phase. Excessive recruitment of pro-inflammatory myeloid cell is attributed to the prolonged expression of pro-inflammatory cytokines at the wound site, altering the local wound microenvironment (98–100). Cellular senescence significantly prolongs the inflammatory phase of chronic wounds in several ways. Firstly, senescent cells prolong the inflammatory state of chronic wounds by secreting inflammatory cytokines, chemokines, and proteases through the SASP. These factors not only continuously activate immune cells but also impair the function of surrounding healthy cells. Secondly, SASP factors disrupt normal cellular signaling, affecting the proliferation, migration, and apoptosis of cells involved in wound healing. This disruption prevents the transition from the inflammatory phase to the proliferative phase, thus extending the duration of inflammation. Additionally, senescent cells hinder the initiation and progression of anti-inflammatory responses, causing persistent inflammation. They also affect the function of immune cells, leading to ineffective clearance of inflammatory factors and cellular debris from the wound site. Lastly, senescent cells alter the local microenvironment, increasing the concentration of inflammatory factors, which further exacerbates chronic inflammation and impedes the overall healing process. In previous studies, the myeloid cell phenotypes during wound healing in healthy and healing-impaired diabetic mice were characterized by *Joshi* et al. They discovered a significantly higher number of immune cells in the advanced stages of wounds, which sheds light on the diversity of myeloid cells and draws attention to the aberrant inflammatory response associated with poor wound healing (101). Myeloid cell intrinsic factors may be a major driver in this process (101). Other previous studies have found that excessive infiltration of myeloid cells was found in the late stage of diabetic chronic wounds in mice (102). Alleviating myeloid cell infiltration can effectively accelerate chronic wound healing in mice.

Typically, the proliferation phase of wound healing begins within 2 to 10 days and involves the proliferation and migration of various cell types, such as immune cells and wound repair-related parenchymal cells, critical for wound repair (103). The behavior of wound repair-related parenchymal cells assumes a critical role during this period. Fibroblasts repair wounds by repairing the damaged dermis and remodeling the ECM in DFUs. Fibroblasts play a key role in repairing the damaged dermis and remodeling ECM, while epidermal keratinocytes proliferate at the wound edge, migrate to the surface, and restore the barrier to re-establish homeostasis. This phase includes re-epithelialization and the formation of a new epithelial layer. However, cellular senescence can significantly disrupt the proliferation phase. This persistent inflammation impairs the proliferation and migration of fibroblasts and keratinocytes, which are crucial for repairing the dermis and restoring the epithelial barrier. Additionally, senescent cells interfere with angiogenesis, which is essential for providing nutrients and blood supply to the wound. Senescent cells secrete inflammatory factors through the SASP, leading to chronic inflammation. Firstly, SASP factors can disrupt the function of proliferative cells such as fibroblasts and keratinocytes (104, 105). Secondly, chronic inflammation driven by these SASP factors interferes with normal cellular signaling and growth factor activity, impairing the proliferation and migration of cells needed for wound closure (18). Lastly, senescent cells hinder angiogenesis and collagen deposition, both critical for effective wound repair (106). In summary, cellular senescence prolongs the inflammatory state, thereby delaying progression to the proliferative phase and impeding the overall healing of chronic wounds.

## 4 The relationship between different types of chronic wound and cellular senescence

Clinically, chronic wound is defined as one that fails to heal and form intact skin within 1-3 months after injury (1). According to statistics, a large number of patients currently suffer from chronic non-healing wounds that resist healing and require expensive treatment globally, such as DFUs, venous leg ulcers (VLUs), wounds in the elderly, Pressure ulcers (PUs) and Arterial wounds. In addition, chronic wounds are often accompanied by repeated infections and inflammation, manifested as clinical signs of redness, swelling, heat, and pain, which brings great pain to patients and imposes a burden on families and countries worldwide. Therefore, it is essential to take effective measures to promote wound healing. Epidemiological research has found that many chronic wounds do not show improvement after standard care.

### 4.1 Diabetic foot ulcers

#### 4.1.1 The definition and discovery of DFUs

DFUs are a serious complication of diabetes and are a typical chronic wound with a high prevalence and disability rate (107). Approximately 15–25% of diabetic patients are at risk of developing DFUs throughout their lifetime (108) and 84% of them may require lower limb amputations (109). The pathogenesis of DFUs remains unclear. The non-healing of DFUs can be attributed to several factors: (1) chronically high blood glucose levels, resulting in vascular damage or incapacity in the wound tissue bed; (2) recurrent infections, leading to damage to the soft tissues of the foot; (3) the poor wound microenvironment slows down the proliferation of vascular endothelial cells, fibroblasts, and keratinocytes, which in turn contributes to the development of diabetic chronic wounds. Prolonged exposure to HG, chronic inflammation, a high ROS load, low growth factor levels, and infection, among many other factors, contribute to the harsh and complex wound microenvironment of DFUs. In addition to standard treatment approaches such as debridement, weight loss, and wound dressing, a series of adjunctive treatments, such as biological tissue engineering skin, recombinant growth factors, and hyperbaric oxygen, have been employed clinically, but their therapeutic effects are limited, and satisfactory treatment outcomes have not been achieved.

#### 4.1.2 Characteristics of DFUs and pathways involved in cellular senescence

Cellular senescence plays a pivotal role in the pathophysiology of DFUs by disrupting normal wound healing through several signaling pathways. In DFUs, hyperglycemia-induced oxidative stress and chronic inflammation lead to the accumulation of senescent cells, particularly in the vascular endothelium and dermal fibroblasts, which are critical for tissue repair. Studies have revealed that the proliferation of resident cells is slowed down in the wound bed of diabetic wounds (110–112). Several studies have demonstrated that epidermal fibroblasts exhibit dysfunction and reduced migration through impaired cell adhesion and integrin subunit function under HG conditions (113, 114). The p38 MAPK pathway is significantly upregulated in senescent cells within DFUs, responding to the sustained oxidative damage and contributing to the secretion of pro-inflammatory cytokines and MMPs, which degrade the ECM and impair wound healing. Additionally, oxidative stress is recognized as one of the main factors contributing to the delayed healing of diabetic wounds. The excessive accumulation of ROS resulting from the increase in serum glucose and the accumulation of advanced glycation end products in diabetes is known to be one of the key factors inducing cellular senescence (115). Diabetes is typically characterized by long-term chronic inflammation. It is well established that inflammatory factors, such as TNF and IL-6, can induce cellular senescence.

### 4.2 Venous leg ulcers

#### 4.2.1 The definition and discovery of VLUs

It is widely recognized that many “venous ulcers” commonly known as “Laolan leg” in China, are the most serious and difficult-to-treat complication caused by chronic venous insufficiency of the lower extremities, as reported in 1868. This is mainly due to the stasis of blood in the distal limbs and the hypoxia of tissues, which leads to skin nutrient disorders and ultimately results in tissue necrosis and the formation of chronic non-healing ulcers. Recent research suggests that microcirculatory abnormalities and inflammatory responses are the underlying mechanisms of VLUs.

#### 4.2.2 Characteristics of VLUs and pathways involved in senescence

VLUs have an increasing incidence 0.3 - 1.33% (116) and prevalence 0.12 - 1.69% (117) in the elderly aged 65 and above (118), resulting in 60% of VLUs developing into chronic wounds (119). Currently, there are four theories related to the pathogenesis of VLUs. Firstly, *Falanga* and *Eaglstrin* et al. suggest that the microenvironment of venous ulcers is not conducive to wound healing (120). They found that endothelial cell decompensation leads to the secretion of macromolecules, which further inhibits the activity of TGF-β and undermines tissue integrity and wound recovery. Wound fluid collected from VLUs inhibits the proliferation of fibroblasts, venous endothelial cells, and keratinocytes *in vitro*. Secondly, *Claudy* et al. propose that the increased activity of leukocytes leads to the secretion of ROS and TNF-α, which increases vascular permeability and induces the deposition of pericapillary fibrin. Thirdly, in 1982, *Browse* and *Burnand* et al. suggested that venous hypertension causes the dilation of endothelial pores, allowing the escape of macromolecules (primarily fibrinogen) into the interstitial fluid. Insoluble fibrin complexes form deposits and create a barrier around the capillaries, promoting cell death and ulceration (121). Fourthly, in 1988, *Coleridge Smith* et al. proposed that the increased pressure in the venous system leads to a decrease in capillary perfusion pressure, and the decrease in capillary flux is sufficient to cause the trapping of leukocytes. Leukocytes then release toxic oxygen metabolites and proteolytic enzymes, which cause capillary damage and make the capillaries more permeable to macromolecules. Increased permeability may lead to the extravasation of fibrinogen and other plasma proteins, resulting in the formation of a fibrin cuff (122). The downstream signaling pathways of venous ulcer fibroblasts are altered, involving MAPK and the early SMAD pathway, by reducing the expression of Type II receptors, which decreases cellular proliferation (123).

### 4.3 Chronic wounds of the elderly

#### 4.3.1 The definition and discovery of chronic wounds in the elderly

Chronic wounds in the elderly are currently poorly characterized. Many factors can influence wound healing in older individuals. A study in the UK using the General Practice Research Database revealed that the incidence of VLUs is three to four times higher after 80 years of age compared to 65–70 years of age (117). According to the latest census data from the United Nations Population Division, there are over 720 million people aged 65 and above, accounting for more than 9% of the world’s population, and these numbers are expected to increase even more rapidly in the next decade (124). The National Institutes of Health reports that aging skin heals more slowly than younger skin, with wounds sometimes taking up to four times longer to heal in the elderly (125). Thinning of the outer skin and more fragile blood vessels are a normal part of the aging process, leading to bruising and bleeding beneath the skin. The subcutaneous fat layer also thins, providing less padding to prevent injury. In a diabetic burn model, aged mice exhibit delayed wound healing due to decreased expression of HIF-1 (126). The capacity for wound healing diminishes with age in many tissue types and organs (127, 128).

#### 4.3.2 Characteristics and pathways of cellular senescence involved in chronic wounds in the elderly

**Figure 6:**
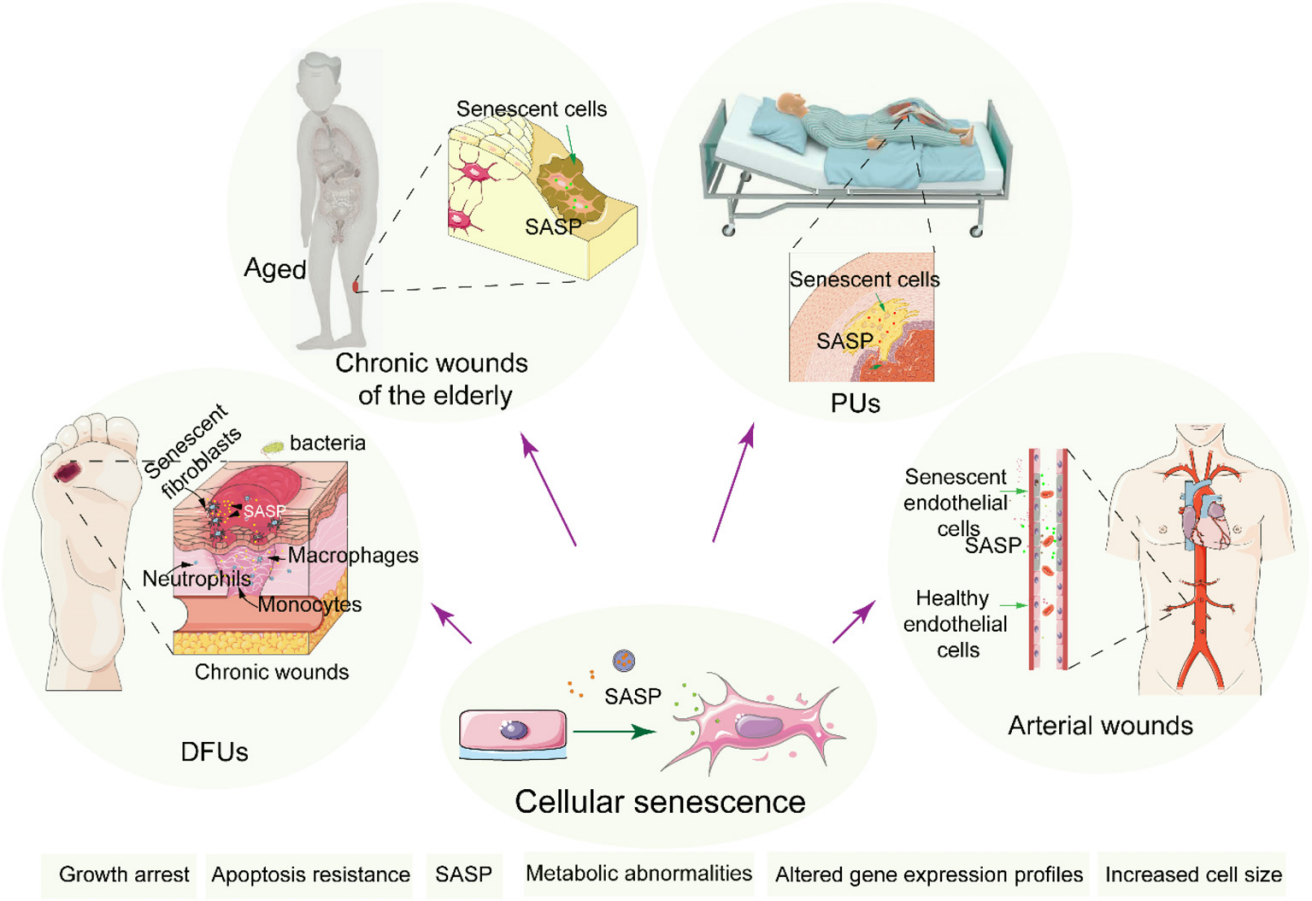
The relationship of cellular senescence and chronic wounds. Cellular senescence in chronic wound cases such as DFUs, chronic wounds of the elderly, PUs and Arterial wounds. Senescent cells accumulate induced by stressors at the wound site, promoting prolonged inflammation and impaired healing, thereby delaying recovery and increasing complications.

The process of wound healing is compromised in the elderly, increasing susceptibility to infections, which puts the elderly at a high risk of developing chronic wounds. Alterations in growth factors, nutritional status, dysfunction of ECM remodeling, and persistent inflammatory response are considered important pathological factors affecting the wound healing of the elderly. In aged individuals, senescent cells accumulate in the skin and vasculature due to repeated exposure to stressors like oxidative damage and diminished regenerative capacity. This accumulation of senescent cells contributes to the chronicity of wounds through several key signaling pathways. Inflammation is a low-grade inflammatory state associated with aging and is considered a biomarker of biological aging and accelerated aging (129, 130). Resident cells within the tissue bed show reduced proliferation and a morphology resembling that of senescent cells (**Figure 6**). Moreover, previous studies have found that the expression and activity of proteases increase in aged individuals, which is consistent with the *in vitro* experimental findings of increased matrix metalloproteinase (MMP) activity in rat wound tissue beds (131, 132). These factors result in exacerbated inflammatory situation and poor microenvironment in the skin wound that is not conducive to healing. Additionally, the properties of the ECM of the skin change dramatically over time (133). Hormone has good prospects in the field of anti-aging (134). A systematic review published in JAMA in 2017 and 2022 demonstrated the use of hormone therapy for the primary prevention of chronic conditions in postmenopausal women (135, 136). Studies have shown that hormonal status plays a critical role in cutaneous wound healing associated with an increase in TGF-β1 levels (137).

### 4.4 Pressure ulcers

#### 4.4.1 The features and discovery of PUs

PUs, also called pressure sores, are localized injuries to the skin and underlying tissues caused by prolonged pressure, typically over bony prominences. Affecting approximately 2 - 3% of hospitalized patients, with even higher rates in long-term care settings, these ulcers are characterized by well-defined lesions ranging from superficial redness to deep tissue damage, often with necrosis and minimal exudate (6). Pathologically, they result from sustained pressure that leads to ischemia, tissue necrosis, and impaired blood flow, primarily due to pressure-induced occlusion of blood vessels, which disrupts oxygen and nutrient delivery. Treatment of pressure ulcers includes: management of local and distant infections, removal of dead tissue, maintenance of a moist environment to promote wound healing, and possible surgery. Although there are many surgical procedures available to treat PUs, surgical flap coverage has not been established as the gold standard for the treatment of PUs (138). Current treatments focus on relieving pressure through regular repositioning, specialized mattresses, and cushions, alongside wound management with advanced dressings and debridement. However, these methods have notable limitations: repositioning is labor-intensive and not always effective in preventing ulcer formation; specialized equipment is costly and not universally accessible; and existing wound care products often fail to address the underlying mechanisms, leading to high recurrence rates and prolonged healing. Therefore, more effective and accessible solutions are urgently needed to improve outcomes for patients at risk of or suffering from PUs.

#### 4.4.2 Characteristics and pathways of cellular senescence involved in PUs

Emerging evidence suggests that the progression of PUs is intricately linked to cellular senescence, particularly through the activation of specific signaling pathways that modulate inflammatory responses and tissue repair (139). The mTOR pathway, a master regulator of cellular metabolism and growth, has been implicated in the promotion of SASP factors in PUs pathology. Inhibition of mTOR has been shown to enhance autophagy, reducing the accumulation of damaged organelles and proteins, thereby mitigating cellular senescence and improving wound healing outcomes (140). Another critical pathway is the p38 MAPK pathway, which is activated in response to various stress signals, including oxidative stress and DNA damage, common in chronic wounds (141). Activation of p38 MAPK contributes to the stabilization of SASP factors and perpetuates the inflammatory environment, leading to impaired healing and tissue degradation (142). Additionally, the NF-ΚB pathway is a key mediator of inflammation in PUs, with persistent activation resulting from SASP factors further exacerbating tissue damage and delaying wound closure (143). Targeting these pathways, particularly through the use of mTOR inhibitors, p38 MAPK inhibitors, and NF-ΚB modulators, holds promise for therapeutic strategies aimed at mitigating the effects of cellular senescence and improving outcomes in patients with PUs.

### 4.5 Arterial wounds

#### 4.5.1 The features and discovery of arterial wounds

Arterial wounds, also known as ischemic ulcers, are chronic lesions caused by inadequate blood supply due to arterial occlusion or stenosis. These wounds are prevalent among individuals with peripheral arterial disease, affecting approximately 1 - 2% of the population, particularly older adults and those with diabetes. Clinically, arterial wounds are characterized by a well-defined, punched-out appearance with necrotic tissue and minimal exudate, typically located on the lower extremities. Pathologically, they result from chronic ischemia due to impaired arterial blood flow, leading to tissue hypoxia and necrosis. The prevailing hypothesis suggests that insufficient oxygen and nutrients not only impair tissue repair but also increase susceptibility to infection. Current treatments focus on improving blood flow through surgical interventions such as angioplasty or bypass surgery, alongside wound management with advanced dressings and debridement. However, these treatments face significant challenges: surgical procedures may not always restore adequate blood flow, wound care methods often fail to address underlying vascular issues, and the risk of recurrence remains high. Therefore, more effective strategies that simultaneously improve vascular health and enhance wound healing are critically needed to better manage arterial wounds.

#### 4.5.2 Characteristics and pathways of cellular senescence involved in arterial wounds

The development of arterial wounds, often associated with peripheral artery disease (PAD), is increasingly linked to cellular senescence, which disrupts normal tissue repair processes through various signaling pathways. In arterial wounds, senescent endothelial cells accumulate due to chronic oxidative stress and reduced blood flow, leading to impaired angiogenesis and wound healing. The p38 MAPK pathway, a key responder to stress signals such as hypoxia and oxidative damage, is activated in senescent cells within arterial wounds. This activation promotes the secretion of pro-inflammatory cytokines and matrix metalloproteinases (MMPs), contributing to extracellular matrix degradation and chronic inflammation (144). Furthermore, the TGF-β/SMAD pathway, which is involved in fibrosis and tissue remodeling, is upregulated in senescent cells, leading to excessive extracellular matrix deposition and fibrosis, further hindering wound healing (145, 146). The NF-ΚB pathway, similarly to its role in other chronic wounds, perpetuates inflammation in arterial wounds by sustaining the SASP, thereby exacerbating tissue damage and delaying repair (147). In summary, therapeutic strategies that target these pathways, such as p38 MAPK inhibitors, TGF-β antagonists, and NF-ΚB modulators, might potentially mitigate the effects of cellular senescence and promote healing in arterial wounds.

## 5 Treatment strategy for chronic wounds

Debridement therapy is the basic local treatment method for DFUs, including sharp instrument debridement, surgical debridement, ultrasonic debridement, and other methods to improve the wound status of DFUs. Timely debridement according to the gangrene condition, removal of necrotic, ischemic, and infected gangrene, and leaving a clean and viable tissue bed to support the healing process. The principle of debridement is that dry gangrene should not be removed prematurely, and infected gangrene should be removed immediately. Debridement is beneficial to the formation of granulation tissue, reducing the healing time and the bacterial load on the wound surface.

### 5.1 Anti-aging drugs and chronic wounds

Cutaneous wounds are one of the most common soft tissue injuries and are particularly difficult to heal in aging. *Zhao* et al. found that chronic topical administration of metformin and resveratrol can accelerate wound healing with improved epidermis, hair follicles, and collagen deposition in young rodents, and metformin has a more profound effect (148). *Mahendra* et al. extracted bioactive compounds from the sky fruit of Swietenia macrophylla, which can absorb the UVB range and some parts of the UVB wavelength (149). *In vitro* scratch experiments found that sky fruit can significantly improve cellular proliferation, migration, and wound closure. Rapamycin has been widely used for decades to treat cancer and prevent organ transplant rejection. However, in recent years, it has gained attention for its potential to extend life. Experimental results on various animal models indicate that administering rapamycin can enhance the health of older animals, particularly females, achieving a more substantial life-extending effect. The latest clinical data reveal that short-term use of rapamycin can activate the type 1 interferon pathway and enhance immune function in the elderly. *Partridge* et al. employed Drosophila and mice as models to elucidate the function of S6 protein kinase in the process of rapamycin regulating lifespan and immune system aging, and identified the endolysosome system as a novel cellular mechanism in the aging process, which plays a significant role (58).

### 5.2 Growth factor and stem cell therapy

Cellular senescence refers to as irreversible growth arrest that occurs after exposure to stress, which contributes to tissue dysfunction by inducing paracrine senescence, stem cell dysfunction and chronic inflammation. Growth factors and cytokines play a vital role in cell proliferation, migration, differentiation, and metabolism. Both *in vivo* and *in vitro* experiments have found that there are disorders of GFs in both acute and chronic non-healing wounds (150–152). A study has found that EGF accelerates the proliferation and migration of many types of cells, including fibroblasts, keratinocytes, and endothelial cells (153). *Chen* et al. reported therapeutic effects of conditioned medium from human umbilical cord mesenchymal stem cells (uMSC-CM) (154). They found that uMSC-CM was effective in mitigating the progression of radiation ulcers by inhibiting cellular senescence. Studies have found that the reduction of growth factors in wound tissue bed is a key factor affecting the healing process of diabetic wounds, and local application of exogenous recombinant growth factors can improve the efficiency of wound closure, such as such as EGF, PDGF, FGF, VEGF, TGF-β (155, 156). However, it also has certain limitations. The continuous exudation environment is not conducive to the residence of the drug, and the exudate contains various proteases and polypeptide enzymes, which can easily make the drug ineffective. Patients with DFUs exceeding 50% are not sensitive to growth factor treatment (157, 158). In addition, there is still some controversy about its safety. In the current clinical treatment, growth factors are advocated as adjuvant therapy on the basis of debridement.

### 5.3 Anti-inflammatory and chronic wounds

Infection is one of the main factors in the difficult healing of DFU wounds. Patients have a very limited ability to clear pathogenic bacteria through autoimmunity, resulting in an increased amputation rate and mortality of affected limbs. The current systemic antibiotic treatment has a positive effect on DFU co-infection, but the damage to the microcirculation leads to the drug’s inability to fully penetrate into the infected wound, which reduces its efficacy. Long-term use is likely to cause acquired drug resistance. According to the “Antibiotic Resistance Threat Report” (http://www.cdc.gov/) published in the United States in 2019, about 2.8 million people in the United States have developed drug resistance due to overdoses of antibiotics causing infection. In addition, nano-silver particles are currently used clinically for anti-infection treatment, but their biocompatibility is poor, and there is a tissue accumulation effect, causing toxicity in the digestive system (159), respiratory system (160) and central nervous system (161). Therefore, it is necessary to develop next-generation antibacterial drugs or strategies to solve the problem of DFU wound infection.

### 5.4 ROS scavenging and chronic wounds

There are different reasons for the formation of chronic, non-healing wounds. However, current research believes that they all have one of the same characteristics: excessive ROS are produced in the wound microenvironment, which sequesters the wound’s healing. Therefore, ROS scavenging is an effective strategy for chronic wounds.

### 5.5 Advanced wound dressings and chronic wounds

For cutaneous wounds, aberrant inflammation, elevated ROS levels, and reduced growth factor expression collectively contribute to impaired neovascularization and cell proliferation, increased cellular senescence, and heightened apoptosis, ultimately leading to delayed wound healing. Thus, identifying an effective medical dressing to enhance chronic wound healing has become an urgent and significant medical challenge. Wound dressings are classified into four primary categories: passive, interactive, advanced, and bioactive. It is estimated that the global market for wound dressing materials will reach $20.4 billion by 2021 (162). The treatment of chronic wounds has long been problematic, with existing dressings often proving ineffective. However, advancements in biomaterials have led to the development of wound dressings that show promising results (162, 163). *Mohamed. A* et al. found a polyelectrolyte wound dressing consisting of chitosan, hyaluronan, and phosphatidylcholine dihydroquercetin, which exert antioxidant and antibacterial activity (164). *He* et al. found a functional chitosan-based hydrogel that serves as a drug delivery system and as a wound dressing to deliver growth factor and antibacterial agents, accelerating wound healing, revascularization, and collagen deposition (165, 166). Compared with the control group, the experimental group has a structure almost the same as the original skin and the largest number of newly formed blood vessels and hair follicles.

Tissue-engineered products have been applied to chronic or non-healing wounds and achieved good results in recent years. They accelerate wound closure by providing growth factors and ECM to the wound microenvironment. The relationship between chronic wounds and prematurely senescent fibroblasts remains to be elucidated. A satisfying wound dressing can provide an optimal biological environment for wound healing. MMP9, a gelatinase abundantly expressed in the dermis after wounding, plays a pivotal role in ECM degradation and tissue remodeling during wound healing (167, 168).

Long-term infection is one of the important factors that make DFUs difficult to heal. In the field of anti-infectives, *Wang* et al. found an engineered bioactive, self-healing antibacterial exosome hydrogel that can promote chronic diabetic wound healing and complete skin regeneration in a mouse model (169). Similarly, it was also reported that a surfactin-reinforced gelatin methacrylate hydrogel accelerates diabetic wound healing (170). Recent studies have found that metal nanoparticle-based materials can reduce the infection of diabetic wounds (171). Researchers found that the combination of antibacterial nanoparticles (such as silver nanoparticles, gold nanoparticles, or copper nanoparticles) with a polymeric matrix could inhibit bacterial growth and speed the healing process of a chronic wound (172, 173).

## 6 Summary

This review explores the link between cellular senescence and chronic wounds. Despite significant efforts to develop effective treatments for non-healing wounds, a definitive solution remains elusive. The review highlights the importance of understanding cellular senescence, senescence-associated secretomes, and their interactions with the tissue microenvironment in the repair of chronic wounds. Targeting senescent cells may accelerate wound healing. It suggests that removing senescent cells from the tissue microenvironment could be a promising approach to enhance chronic wound healing. Nonetheless, a major limitation is the ongoing debate about whether aging exerts a positive or negative regulatory effect on the wound healing process. Targeting cellular senescence holds significant promise as a strategy for improving chronic wound healing. However, to unlock the full potential of this approach, it is crucial to expand research efforts in both basic science and clinical applications. Continued exploration in these areas is expected to bridge the gap between theoretical understanding and practical treatment, paving the way for more effective therapies for chronic wounds.
